# Leveraging active learning-enhanced machine-learned interatomic potential for efficient infrared spectra prediction

**DOI:** 10.1038/s41524-025-01827-8

**Published:** 2025-10-29

**Authors:** Nitik Bhatia, Patrick Rinke, Ondřej Krejčí

**Affiliations:** 1 Department of Physics, Technical University of Munich, Garching, Germany; 2 Department of Applied Physics, Aalto University, AALTO, Finland; 3 Atomistic Modelling Center, Munich Data Science Institute, Technical University of Munich, Garching, Germany; 4 Munich Center for Machine Learning (MCML), Munich, Germany; 5 Department of Mechanical and Materials Engineering, Turku, Finland

**Keywords:** Heterogeneous catalysis, Organic chemistry, Theoretical chemistry, Atomistic models, Computational methods, Characterization and analytical techniques, Characterization and analytical techniques, Infrared spectroscopy

## Abstract

Infrared (IR) spectroscopy is a pivotal analytical tool as it provides real-time molecular insight into material structures and enables the observation of reaction intermediates in situ. However, interpreting IR spectra often requires high-fidelity simulations, such as density functional theory based ab-initio molecular dynamics, which are computationally expensive and therefore limited in the tractable system size and complexity. In this work, we present a novel active learning-based framework, implemented in the open-source software package PALIRS, for efficiently predicting the IR spectra of small catalytically relevant organic molecules. PALIRS leverages active learning to train a machine-learned interatomic potential, which is then used for machine learning-assisted molecular dynamics simulations to calculate IR spectra. PALIRS reproduces IR spectra computed with ab-initio molecular dynamics accurately at a fraction of the computational cost. PALIRS further agrees well with available experimental data not only for IR peak positions but also for their amplitudes. This advancement with PALIRS enables high-throughput prediction of IR spectra, facilitating the exploration of larger and more intricate catalytic systems and aiding the identification of novel reaction pathways.

## Introduction

Infrared (IR) spectroscopy has become an important analytical technique for the identification and characterization of chemical substances^1,2^. Its applications span a multitude of disciplines, including chemistry, physics, biology, astrochemistry, astrophysics, and material sciences, where precise structural characterization is essential.

IR spectroscopy has been widely utilized to investigate gas-phase molecules, liquids, crystals, semicrystalline materials, amorphous solids, and interfaces such as solid/liquid, solid/solid, and solid/gas^3–7^. In catalysis, IR spectroscopy is particularly advantageous for probing reactions in situ, enabling the identification of reaction intermediates and active sites, thus providing insight that facilitate the development of more efficient catalysts^8,9^. Through these diverse applications, IR spectroscopy not only characterizes material structures but also elucidates the relationships between microscopic structures and macroscopic properties, thereby advancing both fundamental and applied research^10,11^.

However, the interpretation of experimental IR spectra and the accurate identification of molecular species remain challenging due to peak shifts and intensity variations induced by interactions with neighboring species, as well as *spectral congestion* (also known as *interference*) arising from overlapping vibrational signals^12,13^. Consequently, theoretical calculations using quantum mechanical methods are often necessary to gain deeper insight and to ensure reliable interpretations^14–20^.

The *harmonic approximation* is the simplest way to compute IR spectra. In the harmonic approximation, vibrational frequencies are given by the second derivative of the potential energy, e.g., computed by density functional theory (DFT). The harmonic approximation, based on the assumption of a harmonic quantum oscillator, takes into account basic quantum effects. However, it completely neglects anharmonic effects and more complex quantum effects, like tunneling^21^. These are crucial for accurately reproducing peak positions and the overall spectral shape^17^. On the contrary, a fully quantum mechanical framework remains challenging due to the exponential scaling of the time-dependent many-body Schrödinger equation, limiting exact solutions to very small systems^22^. The approximative approaches taking into account both, quantum and anharmonic effects can be separated to three classes: First class, containing methods, such as *vibrational self-consistent field*, and *vibrational configuration interaction* that solve the vibrational Schrödinger equation^22^. The second class consist of Semiclassical approaches, such as the *semiclassical initial value representation*^23^ and *Gaussian wavepacket* methods^24^, incorporate quantum effects into classical trajectories at reduced computational cost. Another important class consists of quasiclassical trajectory-based methods, with *imaginary-time path-integral techniques*^22^ such as *centroid molecular dynamics*^25^ and *ring polymer molecular dynamics*^26^. These approximative quantum approaches, are still too computationally expensive for high-throughput calculations.

Ab-initio molecular dynamics (AIMD),^17,18^ which treats nuclei as classical particles, provides a tractable trade-off between accuracy and computational cost, since it naturally includes anharmonicity through the dynamics on the potential energy surface (PES). In this framework, changes of the molecular dipole moment are recorded over time to construct the IR spectrum, thereby overcoming the main limitations of the harmonic approximation^17,18^. A key limitation of AIMD, however, is that nuclei are treated as classical particles, and thus quantum nuclear effects are completely neglected. In contrast, the recently developed *TePIGS* approach^27,28^ builds on AIMD, but augments it with a quantum thermostat and postprocessing steps, thereby incorporating part of the quantum nuclear effects while retaining anharmonic contributions. However, for both methods, the underlying AIMD is computationally demanding, because long molecular dynamics (MD) trajectories are needed for accurate predictions of the peak positions and their amplitudes. Methods that can predict IR spectra accurately and efficiently are therefore urgently required.

Recent advancements in machine-learned interatomic potentials (MLIPs) make MLIPs a strong contender for accelerating or entirely replacing AIMD IR calculations. MLIPs learn the potential energy and interatomic forces from quantum mechanical calculations, typically DFT^29–38^. If sufficiently trained, MLIPs provide accurate energies and forces, and thus MD trajectories, and combined with recent additions of dipole moment predictions^39–49^ unlock vibrational IR spectra calculations three orders of magnitude faster than AIMD simulations^41,50–54^.

However, the development of accurate MLIP-based frameworks for IR spectra predictions requires high-quality training datasets. Constructing such datasets is one of the most time- and cost-intensive aspects of MLIP development, as the data must capture the relevant interatomic interactions while its generation must not exceed computational budgets^55–57^. Conventional data generation methods often involve exhaustive sampling, leading to large datasets with redundant information, increasing computational costs without necessarily improving model performance. One promising solution to this challenge is active learning, a method that enables the systematic selection of the most informative data points, thus reducing the computational burden associated with both data generation and training^50,58–66^. By strategically focusing on regions of chemical space where the model’s uncertainty is highest, active learning ensures that the data collected enhances the MLIP’s accuracy and reliability while minimizing redundancy and inefficiencies.

Building on these advancements, we introduce a Python-based Active Learning Code for Infrared Spectroscopy (PALIRS), an active learning framework designed to efficiently construct training datasets for MLIP-based IR spectrum prediction. In this study, we first assess the effectiveness of PALIRS in training MLIPs-based on neural networks (NNs), and compare its capability to explore the configurational space with that of AIMD. Subsequently, we seek to evaluate PALIRS’s ability to generate accurate IR spectra for small organic molecules relevant to catalysis. We assess how well ML-generated spectra compare to AIMD and experimental references, and determine the simulation time required for molecular dynamics-derived IR spectra to reach convergence. Additionally, we address the possibility to predict the temperature dependence of the predicted spectra, analyzing how spectral features evolve with temperature. Finally, we target the extrapolation limits of the full workflow by assessing its performance on molecules both similar and dissimilar to the training set. In doing so, we aim to quantify prediction errors in relation to molecular features and establish the boundaries of our ML model’s generalizability. With this, we aim to demonstrate a proof of concept for our approach, thereby paving the way for its application to other, larger systems investigated using IR spectroscopy.

## Results

### Computational workflow

To perform MD-based IR spectra predictions with MLIPs, we need two main ingredients: (1) MLIPs for accurate machine-learning-assisted molecular dynamics (MLMD) simulations and (2) accurate dipole moment predictions for computing the autocorrelation function and thus the IR spectrum. Therefore, an accurate description of energies, forces, and dipole moments is critical.

**Fig. 1:**
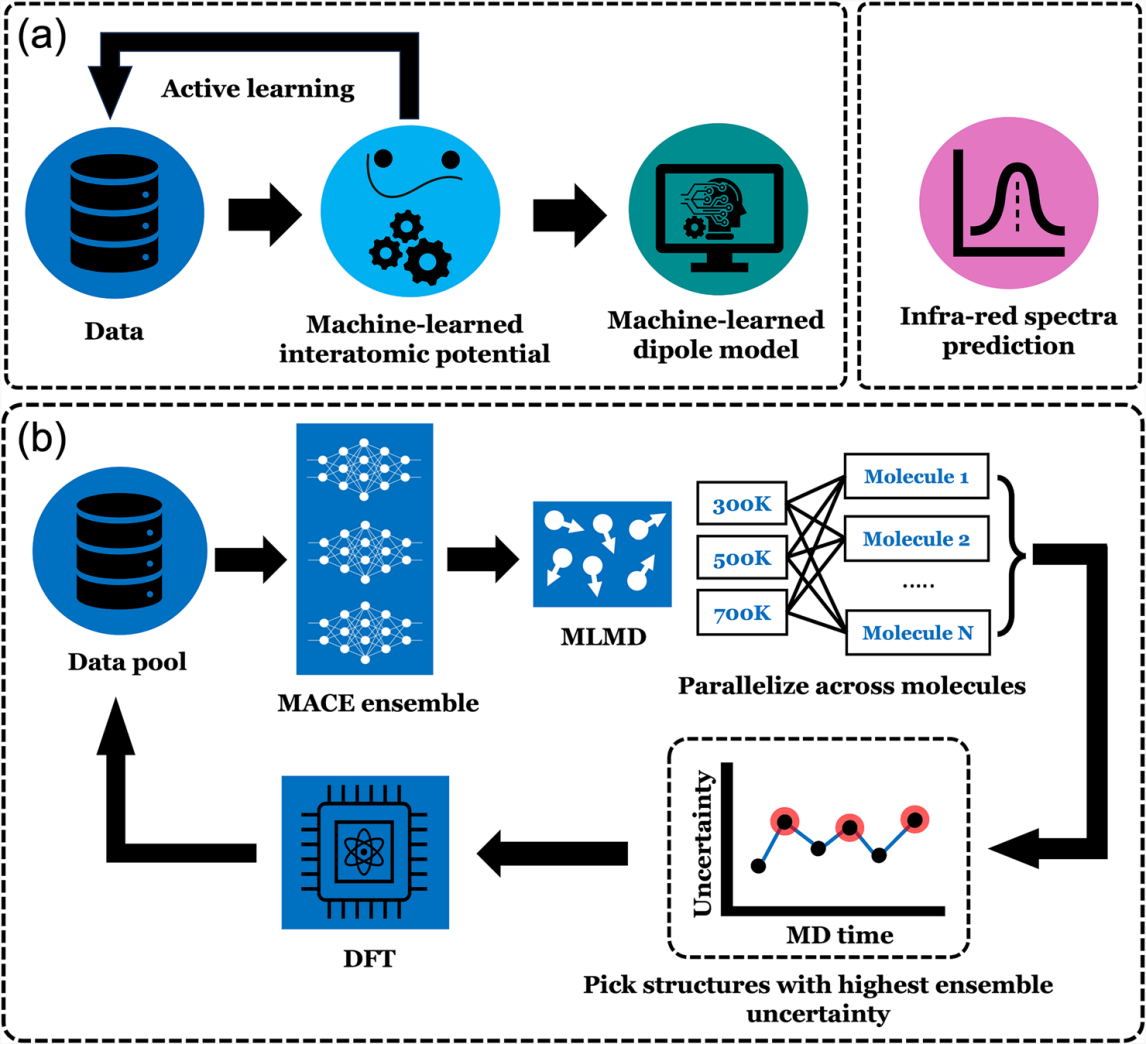
Workflow schematics for machine-learned infrared spectra prediction, as implemented in PALIRS. **a** An overview of the machine learning methodology implemented for predicting infrared spectra from molecular structures. **b** The active learning framework designed to generate high-quality data for training machine-learned interatomic potential (MLIP) models, focusing on the construction of both potential energy surfaces and dipole moment surfaces.

In this study, we introduce a four-step approach to predict the IR spectra of 24 small organic molecules (see Fig. S1 in the Supplementary Information). This workflow is shown in Fig. 1a and consists of the following steps: (1) With the chosen molecules, we prepare an initial dataset of geometries to train the first version of the MLIP, which is then gradually improved through our active learning scheme. After obtaining the final MLIP and dataset, (2) we train an additional ML model specifically to predict the dipole moments for each structure. (3) Using the MLIP for energies and forces, we proceed with MLMD production runs, and calculate dipole moments for all structures along the trajectory with the ML model. Finally, (4) the IR spectra are derived by computing the autocorrelation function of the dipole moments. These key steps are described in detail below. Further information on the implementation in the PALIRS package^67^ and specific settings are provided in the Methods section.

Our four-step approach (Fig. 1a) works in principle with a single MLIP that provides an intrinsic estimate of uncertainty, such as GAP^30^. The uncertainty estimation is a key feature of PALIRS’ active learning strategy. Since NN-based MLIPs like MACE^44,45^ do not come equipped with an intrinsic uncertainty quantification, we employ an ensemble of three MACE models to approximate the uncertainty^68,69^.

**Fig. 2:**
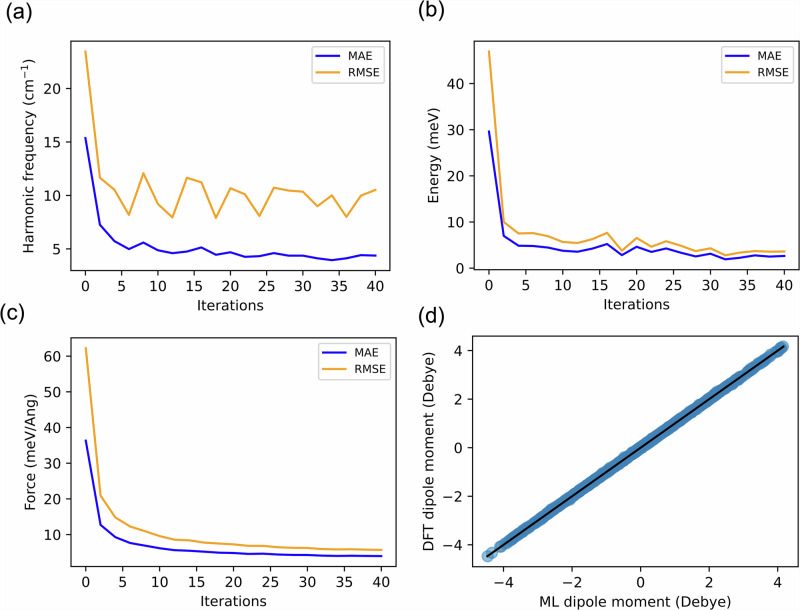
Assessment of MLIP and dipole ML model training and accuracy. Performance of the trained MLIP model, during the active learning procedure: **a** Harmonic frequency, **b** Total energy, **c** Force, evaluated using mean absolute errors (MAEs) and root mean squared errors (RMSEs). The harmonic frequency analysis is based on comparison with all 24 organic molecules, while energy and force evaluations are performed on an independent test set not used in training. **d** Accuracy of the dipole ML model on the same test data as in (**b**, **c**), ensuring consistent performance evaluation across multiple properties.

The initial MACE MLIPs are trained on molecular geometries sampled along the normal vibrational modes^17,31,50,70^ of each molecule. These geometries are obtained from DFT calculations performed with the FHI-aims code^71–74^. While these preliminary MLIPs provide a foundational representation of energies, and forces, their accuracy remains limited due to the relatively small training set, initially consisting of only 2085 structures. The modest performance of these initial models is evident in the learning curve shown in Fig. 2 and will be further discussed in the next section.

To systematically refine the MLIP, we employ an active learning strategy (Fig. 1b) that iteratively expands the training set through MLMD simulations. The acquisition strategy selects molecular configurations with the highest uncertainty in force predictions from each MLMD run, ensuring that the dataset is enriched with the most informative structures while minimizing redundancy. To balance exploration and exploitation during acquisition, the MLMD simulations are performed at three different temperatures: 300 K (low), 500 K (medium), and 700 K (high). Further details on the active learning procedure are provided in the Methods section. The final dataset, after 40 active learning iterations, consists of 16,067 structures, with approximately 600–800 structures per molecule.

It is important to note that the active learning scheme focuses on optimizing energy and force predictions in the MLIPs. A separate ML model, also based on the MACE framework, is specifically trained to predict dipole moments for IR spectra calculations. We refer to this model as the dipole moment model in the following.

### Assessment of active learning performance

To assess MLIP improvement during active learning, we compare its predictions against a predefined test set of harmonic frequencies. These frequencies were obtained as a by-product of the normal mode sampling and include all 24 organic molecules of our study. Harmonic frequencies serve as efficient validation, as they can be rapidly computed with MLIPs and directly compared to DFT reference values. Quantitative metrics such as the mean absolute error (MAE) between MLIP- and DFT-computed harmonic frequencies provide a reliable measure of the model’s accuracy and its progress in describing the studied molecules^50^.

At each iteration, we used the first MLIP in the ensemble to evaluate the harmonic frequencies and quantified its accuracy using the MAE and root mean squared error (RMSE), as shown in Fig. 2a. The initial model, trained solely on molecular configurations from normal mode sampling, starts with an MAE of 15.36 and an RMSE of 23.45 cm^−1^. As active learning progresses, these errors decrease, demonstrating the improvement of the MLIP. After approximately 30 iterations, the MAE reaches a plateau, indicating that the model could no longer be improved by adding more data using the current sampling strategy. We then stop the active learning cycle with a final MAE of 4.37 and RMSE of 10.51 cm^−1^ for the harmonic frequencies.

To evaluate the performance of the MLIP in predicting energies and forces, we constructed a new test dataset generated from a 100 ps MLMD run at 300 K, using the first MLIP model from the final (40th) iteration. Further details are provided in the Methods section. This approach enables a retrospective analysis of how the accuracy of the MLIP evolves throughout the active learning process. The corresponding MAEs and RMSEs are presented in Fig. 2b, c, showing a steady decrease in error with each iteration. In the final iteration, the MLIP achieves a MAE of 2.64 meV and an RMSE of 3.61 meV for energy predictions, and a MAE of 3.96 meV/Å with an RMSE of 5.69 meV/Å for force predictions. These low errors highlight the significant improvement in model accuracy as additional data is incorporated during training.

Subsequently, the dipole ML model is trained on the final dataset obtained through the active learning workflow. The model’s accuracy, validated on the same test dataset used for the energy and force evaluations, is shown in Fig. 2d. The ML model demonstrates strong predictive performance, achieving an MAE of 7.62 and an RMSE of 12.46 mDebye. These results, along with the corresponding errors for energy and force predictions, are summarized in Table 1.

**Table 1:** Final ML model performance on test data

Property	Unit	Test data	
		MAE	RMSE
Energy	meV	2.64	3.61
Force	meV/Ang	3.96	5.69
Dipole moment	mDebye	7.62	12.46

MAE and RMSE stands for mean absolute error and root mean squared error, respectively.

### Configurational and energy space exploration through active learning

**Fig. 3:**
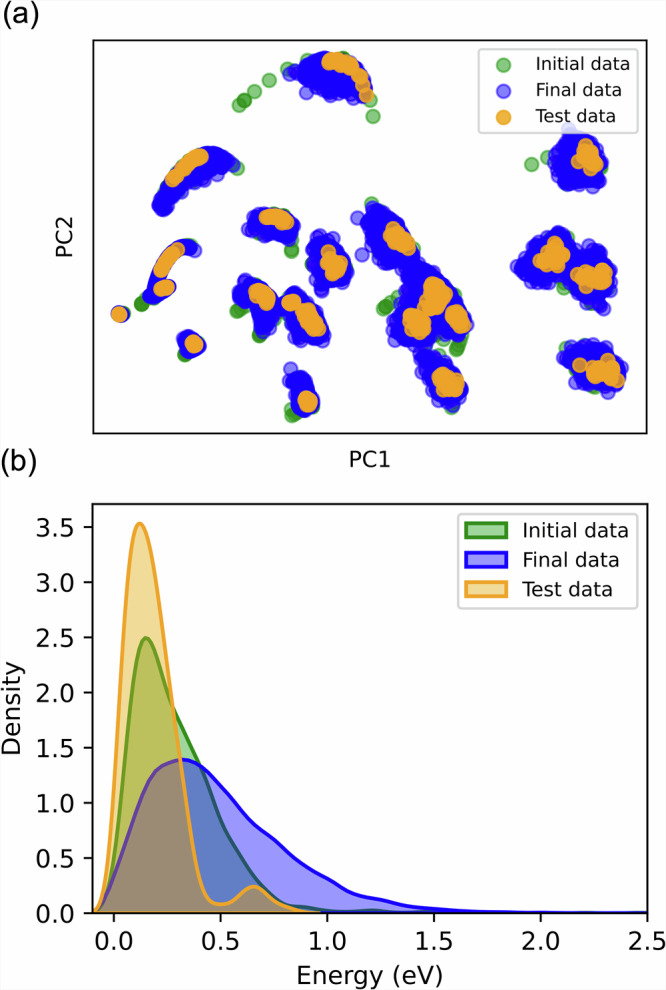
Data distribution analysis. **a** Principal component analysis of the many-body tensor representation for all 24 organic molecules. **b** Energy distribution, with energies calculated by subtracting the optimized structure’s energy. The three data categories are: Initial data (2085 samples, generated via normal mode sampling), final data (16,067 samples, obtained through active learning), and test data (480 samples, produced using the final MLIP).

PALIRS demonstrates high efficiency, characterized by exceptionally low error rates, even when trained on significantly fewer data points than conventional AIMD-based datasets. To investigate the underlying reasons for this performance, we visualized the configurational space using principal component analysis (PCA) applied to the many-body tensor representation (MBTR)^75,76^ (Fig. 3a), and examined the corresponding energy distribution (Fig. 3b).

The PCA plot shows that our dataset of 24 small organic molecules forms several tightly grouped clusters. While some clusters are distinctly separated, many are closely spaced, merging into larger groupings. This indicates that the molecules share structural or chemical similarities, facilitating the MLIP’s ability to learn their properties. The plot also distinguishes initial, training, and test data using different colors, illustrating how the training data gradually expands to cover a broader region of chemical space compared to the sparser initial dataset. This progression, driven by active learning, helps sample underrepresented configurations. The test data is well-distributed across the space, enabling a robust evaluation of model performance.

The energy coverage shown in Fig. 3b is defined as the difference between each configuration’s total energy and that of the corresponding optimized molecule. Initially, the dataset spans a narrow energy window of approximately 1 eV. However, through active learning, the final dataset expands to cover a broader range exceeding 1.5 eV, despite the relatively small number of data points. This wider distribution – still centered near zero—ensures that both low- and high-energy configurations are well-represented, contributing to a more robust and generalizable MLIP.

The test data, derived from an extended MLMD trajectory at 300 K, spans an energy range of about 1 eV, making it well-suited for evaluating the model’s performance across the relevant energy landscape for IR calculations. The broader energy coverage of the final training set ensures that MLMD simulations for IR spectra remain within the domain of the MLIP and dipole models.

Additionally, Fig. S2 (Supplementary Information) compares the configurational space explored by a DFT-based AIMD trajectory for methanol with that sampled via active learning. The results demonstrate that our active learning approach captures a wider energy range and a more diverse configurational space, highlighting its effectiveness in sampling complex molecular environments.

### Infrared spectra calculation and length of dynamical simulation

To compute the IR spectrum, a molecular dynamics (MD) trajectory is generated at a chosen temperature. The spectrum is then obtained by evaluating the autocorrelation function of the time derivative of the dipole moment along the trajectory^17^. The precise formulation and methodological details are provided in the Methods section. However, the reliability of this approach is strongly influenced by the length of the MD simulation, as shorter trajectories can result in noisy or inconsistent spectral features^17^. To address this, we began our study with a systematic analysis aimed at identifying the minimum trajectory length required to achieve spectral convergence.

**Fig. 4:**
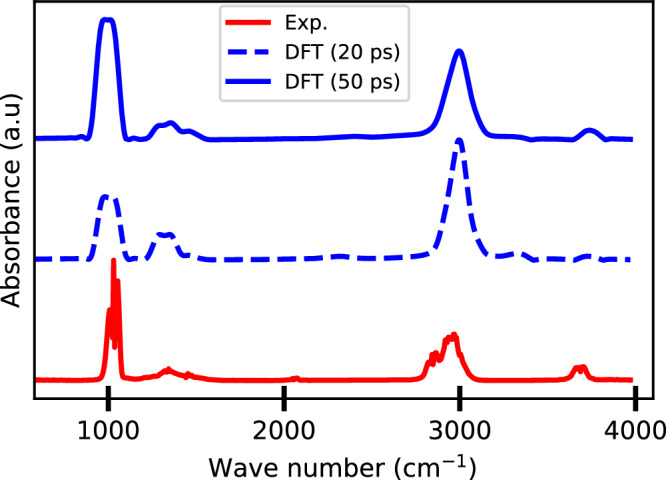
The effect of MD run length on spectra prediction. Comparison of gas-phase methanol spectra obtained by DFT-based AIMD simulations of 20 ps and 50 ps with the experimental spectrum (Exp.) obtained from the NIST database. All calculations and experiments were proceed at 300 K.

Figure 4 presents the IR spectra of gas-phase methanol obtained from DFT-based AIMD simulations with two different trajectory lengths: 20 ps and 50 ps. The spectral peak positions converge by 20 ps, aligning with previous findings^17,77^, suggesting that the key spectral features are already well captured at this timescale. However, the relative peak intensities at 20 ps exhibit an inverse trend compared to experimental spectra, and were also found to vary depending on the initial molecular geometry and velocity distribution. In contrast, the 50 ps simulation yields a spectrum that more closely matches the experimental NIST data^78^. Notably, the experimental peak just above 1000 cm^−1^ is significantly more intense than the one below 3000 cm^−1^–a trend accurately reproduced in the 50 ps simulation. Based on these observations, we adopted 50 ps MD trajectories for all subsequent IR spectra calculations, including both DFT-based AIMD and ML-based predictions using MLMD simulations.

To quantify the similarity between the simulated IR spectra and the experimental data, we use Pearson’s correlation coefficient (PCC) and the Wasserstein distance (WD), as these metrics have proven effective for assessing IR spectra similarity^77^. PCC values range from −1 to 1 (with 1 indicating perfect similarity), while lower WD values indicate closer similarity, with 0 representing perfect alignment. A detailed description of these metrics is provided in the Methods section.

The PCC for the 20 ps simulation, shown in Fig. 4, is 0.68, and increases to 0.73 for the 50 ps simulation. These values align well with previous DFT-based AIMD results^77^. The improvement in PCC with longer simulations underscores the necessity of longer, more computationally demanding runs for accurate IR spectra predictions, reinforcing the efficiency of our approach implemented in PALIRS.

### Performance of PALIRS in predicting infrared spectra

**Fig. 5:**
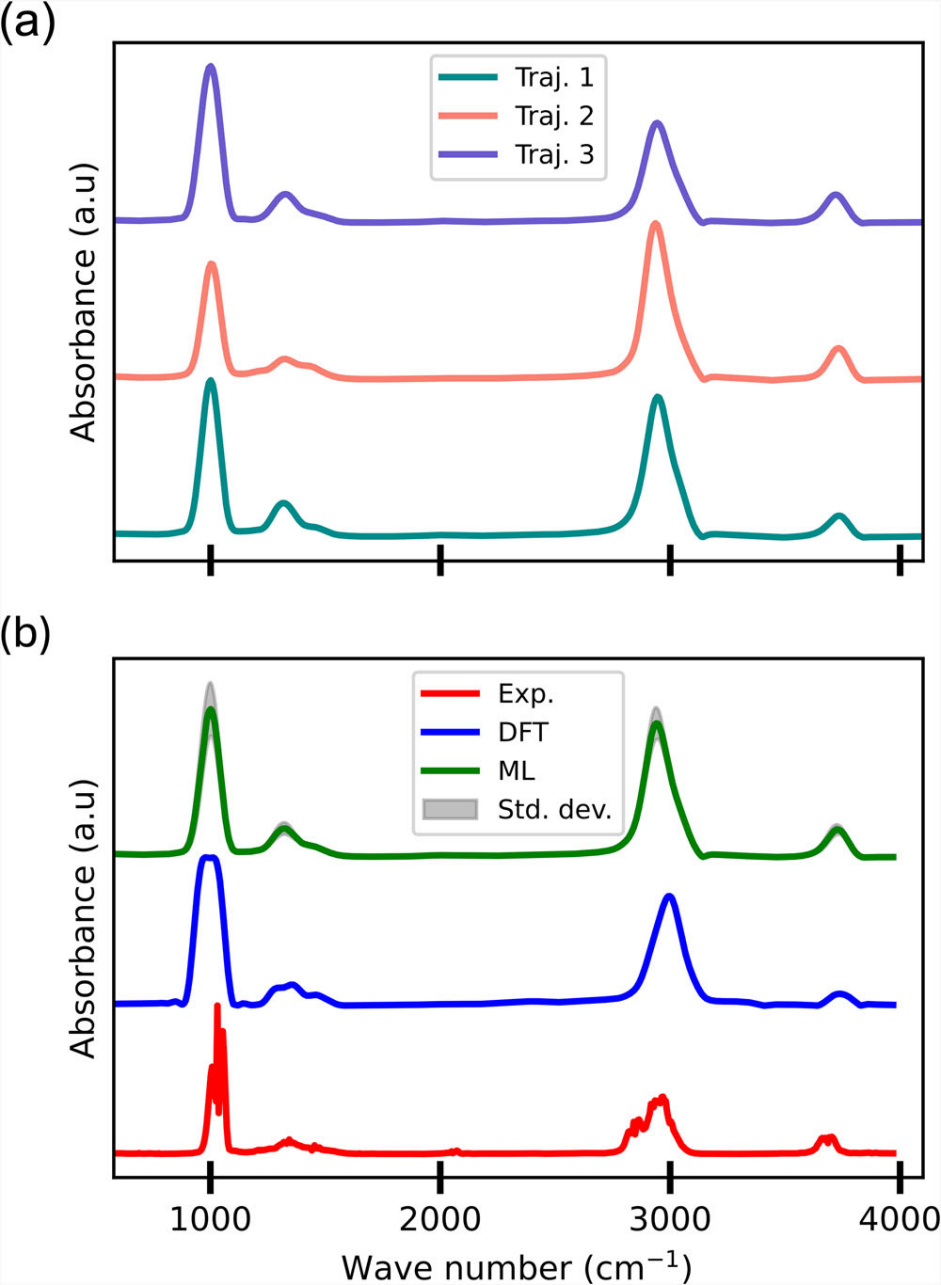
Infrared spectra of methanol in the gas phase at 300 K. **a** Spectra generated using three trajectories (Traj.) from the IR predicting ML models, and **b** a comparison between the experimental spectrum from the NIST database, DFT-based AIMD simulations, and the ML-predicted spectrum. averaged over three trajectories. The standard deviation (Std. dev.) of the ML spectrum is included to represent the uncertainty in the predictions.

With the simulation procedure established and the energy, force, and dipole moment accurately reproduced relative to DFT, we now proceed to the next critical step: evaluating the performance of the trained MLIP and dipole moment model in predicting IR spectra. For the IR spectra prediction, we take advantage of the MLIP ensemble. Specifically, we conduct three separate MD simulations using the MLIP ensemble and the single dipole moment ML model (altogether referred to as ML models). For each MLMD trajectory, an IR spectrum is generated (Fig. 5a), and the final prediction is averaged across the three spectra. Additionally, the standard deviation between the runs is highlighted, providing an indication of the inherent uncertainty in the predictions.

Figure 5b presents a comprehensive comparison of the IR spectra for gas-phase methanol, including the ML-predicted spectrum, the experimental reference from the NIST database^78^, and the DFT-based AIMD spectrum at 300 K. The MLMD and AIMD results show remarkable agreement, with both major and minor peaks aligning closely in terms of position and intensity. When compared to the experimental spectrum, the predicted intensities follow the same overall trend, with only minor shifts observed in peak positions.

The quantitative analysis in Table 2 further confirms the minimal deviation between the DFT and ML results. Notably, it also indicates that the ML predictions align even more closely with the experimental data than the DFT-based AIMD results. This enhanced performance of the ML models can be attributed to the use of three independent MLMD simulations, which collectively sample a broader configurational space than a single DFT-based AIMD run. This more extensive sampling enables the ML-predicted IR spectrum to capture a wider range of molecular configurations, resulting in improved agreement with experimental observations. Overall, the ML models demonstrate a high degree of fidelity in reproducing spectral features, closely matching both experimental and DFT-based spectra.

**Table 2:** Comparison of methanol spectra similarity across methods at 300K

Comparison	PCC	WD
Exp.-DFT	0.73	0.054
DFT-ML	0.91	0.010
Exp.-ML	0.80	0.057

PCC and WD are Pearson’s correlation coefficient and Wasserstein distance, respectively.

The computed IR spectra for the remaining 23 organic molecules are available in the PALIRS repository^67^, while the corresponding PCCs and WDs are provided in the Supplementary Information (Fig. S3). A summary of the statistical analysis is presented in Table 3.

**Table 3:** Quantitative estimation of spectral similarity across all the 24 organic molecules

Comparison	$\overline{{\rm{PCC}}}$	*δ*PCC	$\overline{{\rm{WD}}}$	*δ*WD
Exp. - DFT	0.68	0.22	0.045	0.025
DFT - ML	0.80	0.18	0.026	0.022
Exp. - ML	0.81	0.15	0.029	0.013

The experimental data (Exp.) were obtained from the NIST database. The line above a quantity indicates the mean, while *δ* represents the standard deviation.

Overall, the ML model exhibits excellent accuracy in predicting IR spectra, showing strong agreement with both DFT-based and experimental results. This underscores the efficiency of our approach in significantly reducing computational cost without compromising predictive performance. Leveraging our active learning strategy, fewer than 1000 single-point DFT calculations per molecule were sufficient to generate accurate IR spectra-compared to the over 100,000 required for DFT-based AIMD.

For example, a 50 ps MLMD simulation of methanol produced results comparable to DFT-based AIMD in just 1 h on an NVIDIA Volta V100 GPU, whereas the DFT-based simulation required 107 CPU hours per core on an Intel Xeon Gold 6230. This dramatic speedup not only benefits small molecules like methanol but also scales favorably for larger systems. While MLMD runtimes scale linearly with the number of atoms *N*, i.e., ${\mathcal{O}}(N)$, the computational cost of DFT-based AIMD increases approximately as ${\mathcal{O}}({N}^{3})$. This stark contrast makes MLMD a highly scalable and efficient alternative for simulating larger systems.

### ML model performance across temperature

**Fig. 6:**
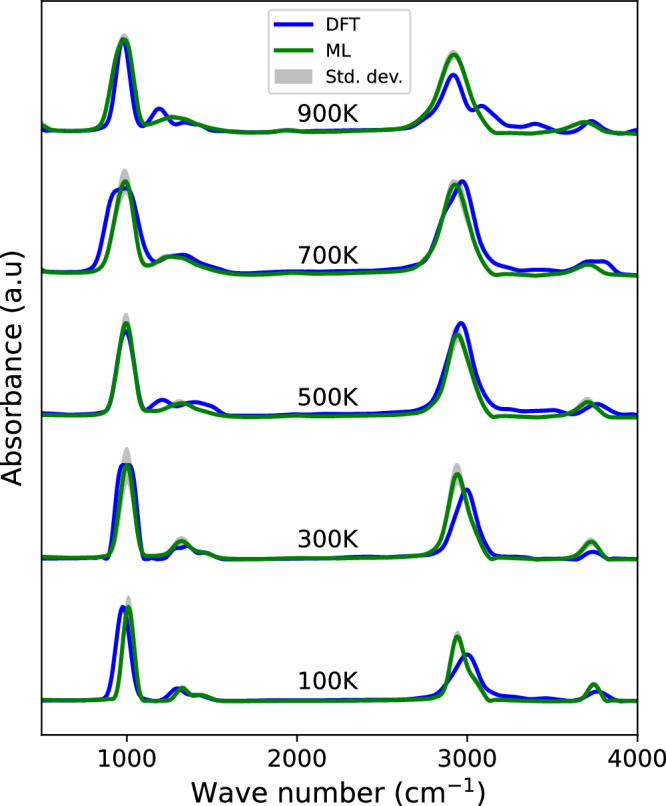
Assessment of the ML model temperature dependence. Infrared spectra of methanol in the gas-phase at five different temperatures (100 K, 300 K, 500 K, 700 K, and 900 K) calculated with the ML models and DFT-based AIMD.

To further test our IR prediction capabilities, we compare the DFT-based AIMD spectra of methanol with the ML-predicted spectra at five different temperatures in Fig. 6. At both low and high temperatures, the band positions are well predicted by the ML models. However, the intensity around 3000 cm^−1^ is slightly overestimated compared to the DFT-based AIMD results. The average PCC and WD values for different temperatures are 0.93 and 0.003, respectively, with standard deviations of 0.046 and 0.001. For detailed values, please refer to the Supplementary Information (Fig. S4). Overall, the results clearly indicate that the ML models have effectively captured the temperature-dependent behavior of the spectra, demonstrating accurate predictions across a wide range of temperatures. An identical analysis has been conducted for ethanol in the gas phase, with the calculated IR spectra and corresponding PCC and WD values also available in the Supplementary Information (Fig. S5), showing even better similarity between the ML and DFT-predicted spectra.

### Assessment of ML model transferability

To evaluate the ML models’ performance beyond the organic species included in the training data, we selected a set of 8 molecules with increasing carbon counts and varying functional groups. Using the same procedure, we compare the average ML-predicted spectra with the experimental data from the NIST database. The full set of 8 molecules and their PCCs and WDs are provided in Fig. S6 in the Supplementary Information, offering a quantitative assessment of the ML models’ transferability.

**Fig. 7:**
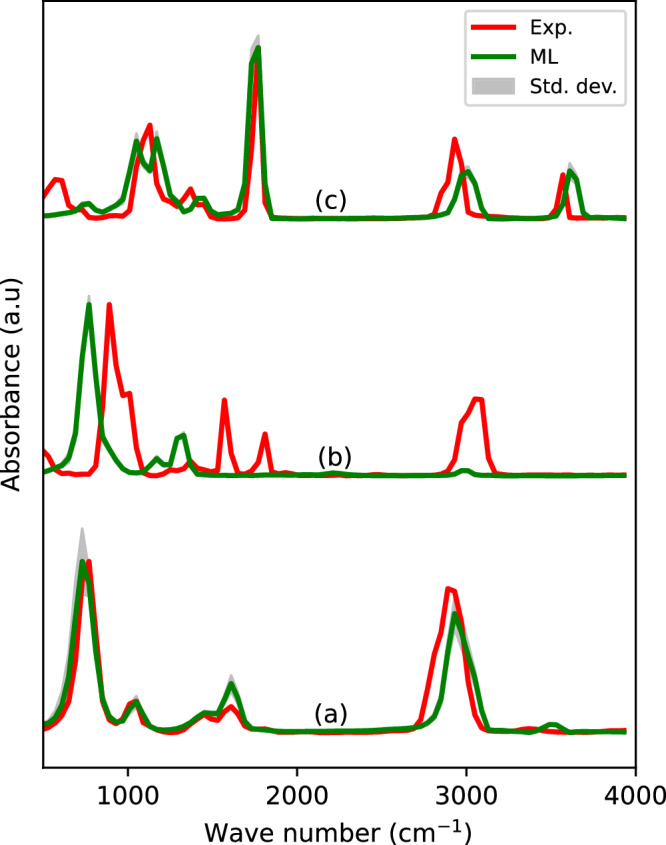
Transferability of ML models in the prediction of infrared spectra in gas-phase. **a** Methyl amine, **b** 1,3-butadiene, **c** pentanoic acid.

In Fig. 7, we present three representative cases illustrating the ML models’ best, worst, and intermediate performance. The best agreement is observed for methyl amine, where both peak positions and intensities closely match the experimental spectrum. In contrast, for 1,3-butadiene, significant deviations in both frequency and intensity patterns are evident. Pentanoic acid shows intermediate agreement, with reasonable alignment in peak positions and as well as in relative intensities. These results highlight a clear dependence of predictive accuracy on the presence of similar chemical environments in the training data, as reflected in the quality of the predicted spectral features.

## Discussion

The MLIPs and the dipole moment ML model demonstrate strong potential for accelerating IR spectrum prediction of small molecules. Our results indicate that our active learning strategy reduces the amount of data needed per molecule by a factor of 10 compared to earlier ML-based approaches^40–43^, and by a factor of 100 compared to conventional AIMD methods, while maintaining good agreement with AIMD reference spectra. Moreover, the training configurations selected by PALIRS effectively cover the configuration space (Fig. 3), underscoring the ability of active learning to focus on physically meaningful variations rather than redundant data points.

Building on these results, the strong qualitative agreement between PALIRS predictions and reference spectra suggests that ML-based models can implicitly capture anharmonic effects-phenomena that are typically difficult and computationally expensive to model using conventional ab initio methods. It should be noted, however, that unlike the quantum and semiclassical approaches discussed in the Introduction, our method does not include quantum nuclear effects^22^, whose incorporation entails significantly higher computational cost. As a result, spectral features arising from phenomena such as combination bands or Fermi resonances may not be captured^79^. However, the average PCC of 0.81 between MLMD spectra and experiment for our 24 molecules shows that the overall agreement is very good, indicating that the quantum effects do not play a substantial role for these systems.

By leveraging MLMD, PALIRS enables spectral convergence through longer trajectories at a fraction of the computational cost of AIMD. This efficiency makes it particularly well-suited for high-throughput evaluation of vibrational spectra, especially for catalytically relevant intermediates^40–43,50–54^.

Given the temperature dependence of vibrational spectra, we assessed PALIRS at various temperatures and found it accurately captures temperature-induced shifts in peak positions and intensities. This highlights the ability of MLMD to capture temperature-dependent spectral changes without explicitly recalculating forces at each temperature using computationally expensive AIMD simulations. However, we also observe that temperature effects introduce subtle discrepancies, particularly in the broadening of certain peaks at higher temperatures. These differences suggest that while MLMD-based IR spectra effectively account for anharmonic effects, further improvements in training set diversity could enhance robustness at extreme conditions.

While PALIRS demonstrates strong performance for small organic molecules represented in the training set, its predictive accuracy diminishes for more complex or chemically distinct systems. For example, the poor performance observed for 1,3-butadiene can be attributed to the limited representation of C=C bonds in the training data, ethene being the only such example, restricting the model’s ability to generalize to similar bonding environments. In contrast, molecules like methyl amine and pentanoic acid, which contain nitrogen and oxygen functional groups that are well-represented in the training data, are predicted with reasonable accuracy. Enhancing the training set to incorporate a wider range of bonding motifs and chemical diversity would help improve generalization. In this context, transfer learning offers a promising pathway to adapt PALIRS for new molecular families.

The limitation in generalization is also reflected in the uncertainty of force predictions during MLMD simulations. For molecules within the training distribution, the maximum force uncertainty across the ensemble of MLIPs remains low, around 10^−5^ eV/Å. However, it increases to 10^−3^ eV/Å for methyl amine and reaches up to 10^−1^ eV/Å for 1,3-butadiene. Despite these elevated uncertainties, the predicted IR spectra remain consistent, indicating that variations in force predictions do not necessarily translate into spectral deviations. This is also evident from the standard deviations shown in Fig. 7.

To clarify the origin of these limitations, we analyzed the relative transferability of the PES versus the dipole moment surface (DMS). To quantify these errors, we generated an additional test set comprising of the 8 molecules employed in the transferability assessment (see Methods for more details). The corresponding errors in energies and forces are reported in Table S1 in the Supplementary Information. For small polar molecules, such as methyl amine, isopropyl alcohol, and acetone, the spectra are predicted accurately, with minor deviations arising primarily from limitations in the PES rather than the DMS. For longer-chain polar molecules, such as pentaneamine and pentanoic acid, the reduced accuracy stems from a combination of both PES and DMS limitations. In contrast, for non-polar molecules with double bonds, like 1,3-butadiene and benzene, the PES is the dominant source of error, while the DMS remains relatively transferable. These observations highlight that improving PES coverage in the training set is critical for enhancing generalization, particularly for chemically distinct or larger systems, while the DMS generally retains reasonable transferability across molecules with similar functional groups.

Lately, pretrained MLIPs on larger datasets, such as MACE-off^49^, are demonstrating growing success of universal MLIPs in atomistic simulations. MACE-off offer MLIPs trained on large and chemically diverse datasets at the *ω*B97M-D3(BJ)/def2-TZVPPD level of theory, using the SPICE dataset^49,80^. In contrast, our PALIRS approach is developed at the PBE level of theory^81^, combined with the Tkatchenko-Scheffler treatment of van der Waals interactions^82^, chosen to remain consistent with widely adopted practices in catalysis-focused studies^77,83–85^. Importantly, the PALIRS framework is not limited to our choice of DFT functional; it can be systematically adapted to train MLIPs across different molecular families and at any level of electronic structure theory.

MACE-OFF MLIPs have showcased high accuracy in computing harmonic IR spectra for diverse molecular datasets^48,49^, but their performance for finite-temperature MD-based IR spectra has not been systematically evaluated. In this context, although PALIRS is primarily optimized for MLMD-predicted IR spectra, a trained MACE model can also be applied within the harmonic approximation, where it shows very good agreement with DFT reference results, as demonstrated in Fig. S7 and Table S2 of the Supplementary Information. It is worth mentioning that the harmonic approximation leads to necessity of frequency rescaling factors, when comparing with experimental spectra.

Thus, our main comparison focuses on finite-temperature MD-based IR spectra, which are more directly relevant to experimental conditions. For this, we computed IR spectra for methanol and ethanol using our ML model and two different versions of MACE-off models, MACE_off23 and MACE_off24, and compared them with experimental spectra from NIST (Fig. S8, Supplementary Information). Quantitative similarity metrics (Table S3, Supplementary Information) show that our ML model consistently achieves higher PCC values than both MACE-off models for both molecules. The MACE-off spectra reproduce the main vibrational features, but exhibit systematic blue-shifts in peak positions compared to experiment, which can be caused by the level of theory or training data. This suggest necessity of thorough testing and consequent fine tuning of universal MLIPs for MD-based IR spectra, while PALIRS approach can be easily adjusted for users’ needs.

In summary, we demonstrated the effectiveness of combining active learning with MLIPs and a dipole moment model for predicting IR spectra of small organic molecules. The PALIRS-trained models achieved accuracy comparable to or better than DFT-based AIMD, while requiring 100 times fewer DFT calculations. A 50 ps MD trajectory was found sufficient for spectral convergence, and the ML models accurately captured temperature-dependent features and generalized well to larger, structurally similar molecules.

Given the relevance of many studied molecules to catalysis, this approach offers a scalable, cost-efficient alternative for high-throughput spectral analysis. We also compared PALIRS with existing pretrained MLIPs, highlighting that while these models are valuable tools, further adaptation is needed for more accurate IR predictions. In contrast, the PALIRS framework provides a general and flexible approach for training MLIPs specifically aimed at MD-based IR spectra.

Future work will therefore focus on expanding the dataset via transfer learning, leveraging universal potential and dipole models where appropriate, and applying the framework to larger and more diverse catalytic systems to support inverse molecular design.

## Methods

### Machine learning models

We trained the equivariant message-passing neural network, MACE^44,45^ and used it to predict total energies and forces. We trained the MACE model with 128 channels, (L=1) equivariant messages, and a cutoff radius of 3.0 Å. The models were structured with two layers, each having a body order of 2, and were trained with MACE version 0.3.5.

In addition, a MACE model was trained to predict dipole moments for infrared spectrum calculations. The model architecture mirrored that of the previously described MACE framework, with the key difference being that the readout function outputs a vector representing the dipole moment for each atom, rather than scalar atomic site energies.

All our MACE training and predictions were performed on NVIDIA Volta V100 GPUs.

### DFT computational details

All DFT calculations were performed using the all-electron numeric-atom-centered orbital code FHI-aims^71–74^. The Perdew-Burke-Ernzerhof exchange-correlation functional (PBE)^81^ was used for the calculations. Further computational settings included the standard FHI-aims tier-1 basis sets and “light" grid settings, the zeroth-order regular approximation to account for scalar relativistic effects^86^, and a Hirshfeld correction term for van der Waals interactions^82^. Structure optimization was carried out using the Broyden-Fletcher-Goldfarb-Shanno (BFGS) minimizer^87^, with a convergence limit of 1 meV/Å for the maximum atomic force amplitude. Geometry optimization and AIMD simulations were performed using the conventional FHI-aims software, while the normal mode sampling and active learning tasks were conducted using the Atomic Simulation Environment (ASE)^88^ and the FHI-aims calculator.

### Initial data generation and MLIP model

Our dataset comprises 24 organic molecules containing hydrogen, carbon, nitrogen, and oxygen. Each molecule has up to two carbon atoms (Fig. S1). Their starting geometries were extracted from the QM9 dataset^89^ and subsequently re-optimized at the DFT level. Starting from the optimized geometries, static calculations based on the harmonic oscillator approximation were performed, and the structures were sampled along the normal modes^17,31,50,70^. Each normal mode corresponds to a distinct way of displacing the structure from its local minimum energy configuration, with all atoms oscillating at the same frequency (refer to Fig. S9 in Supplementary Information)^17,31,50^. We used the first 10 geometries from each of the normal modes sampled by ASE with its default settings^88^.

Single-point DFT calculations were performed on the sampled data to compute total energies, forces, and dipole moments. In the end, 2085 structures (50–200 structures per molecule) were collected and used to train the initial ensemble of MACE MLIP models to start the active learning workflow. The entire dataset was split into a training subset and a test subset with a ratio of 80:20 during initial and subsequent training. The MLIP ensemble was trained on the same dataset. The ensemble MLIPs differ only in the random seed parameter, which controls the initialization of model weights during MACE training.

### Active learning workflow

The ensemble of MACE MLIPs enables the identification of structures with high uncertainties^68,69^, which is a key concept of our active learning workflow. The ensemble MLIPs are iteratively improved by augmenting the dataset with the 15 structures of each molecule with the highest uncertainty in each iteration. This selection process leverages three MLMD simulations conducted at 300, 500, and 700 K, extracting the five most uncertain structures from each temperature. The use of low, intermediate, and high temperatures strikes an optimal balance between exploring new regions of the chemical space and effectively exploiting known regions. A sanity check is implemented that monitors the forces of configurations during the MLMD simulations. For each configuration, we calculate a relative force error as the root of the variance of the predicted forces normalized by the force magnitudes, with a small regularization factor added to prevent division by zero. If this relative error exceeds a threshold of 0.5, the MD simulation is immediately terminated to avoid the generation and propagation of unreliable structures. At each iteration of the active learning cycle, MLMD sampling is performed by continuing the trajectory from the previous iteration, using its final geometry and velocities as the initial conditions.

For the new structures selected by each iteration, we perform single point DFT calculations and add the results to our dataset. After splitting the updated dataset again, all three models are retrained from scratch with different random seeds to remove bias from previous training states, ensures diversity among the models in the committee, and to provide a more reliable estimate of the predictive uncertainty. We repeat this cycle until the estimated error in harmonic frequencies either falls below the selected threshold (5 cm^−1^) or reaches a maximum of 40 iterations.

To accelerate the sampling process on a high-performance computing cluster, the 24 molecules are divided into three batches, each containing 8 molecules. Within each batch, the molecules are sampled in parallel at different temperatures, optimizing the use of computational resources efficiently. Dividing into batches helps balance the workload and prevent bottlenecks in resource allocation, ensuring a more efficient and manageable distribution of computational tasks.

Finally, 600–800 instances of uncertain data are collected per molecule, and the final training data consisted of 16,067 structures. With the final dataset we train the MACE dipole model.

### PALIRS

The PALIRS repository^67^ provides all necessary scripts for initial data generation, setting up the active learning workflow, and the final trained models. It also includes detailed documentation and a concise tutorial to facilitate a seamless workflow setup. While PALIRS is configured for MACE MLIPs and FHI-aims, it can be readily adapted to other ASE-compatible MLIPs and DFT codes.

### Test sets generation

**Fig. 8:**
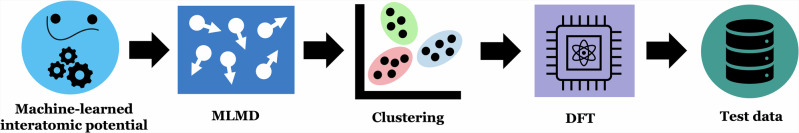
Test data. A streamlined approach for constructing a test dataset, validating the ML models generated at each iteration of the active learning loop.

To evaluate whether the final ensemble of MLIPs can be reliably applied to IR simulations, the first MLIP of the ensemble is used to create a test dataset, as illustrated in Fig. 8. First, a 300 K, 100 ps MLMD simulation is performed for each molecule, from which 2000 atomic structures are uniformly sampled. These structures are then represented using the MBTR descriptor^75^, as implemented in the DScribe library^76^. We use K-means clustering^90^ in the MBTR feature space to select 5 clusters. 4 structures are randomly selected from each cluster, yielding a total of 20 structures per molecule. This clustering-based selection effectively reduces bias and guarantees a broad coverage of the configurational space^66^. In total, the final test dataset consists of 480 structures, and DFT calculations are performed on the selected structures.

The additional test set for transferability prediction evaluation was created the same way, just with 50 structures per molecule, leading to 400 structures in total.

### MD simulations

DFT-based AIMD IR spectra calculations proceed in two stages: First, the system is equilibrated using a Berendsen thermostat with a relaxation time of 0.1 ps^91^, at a specified temperature for 4 ps. Subsequently, a Nosé-Hoover thermostat, with an effective thermostat mass of 4000 cm^−1^ ^92,93^, is employed for a 50 ps run. Only the Nosé-Hoover run is utilized for computing the IR spectra.

All MLMD runs are performed with a Langevin thermostat^94^ with a friction coefficient of 0.01 as implemented in ASE 3.22.1^88^. 5 ps MLMD runs are used during active learning to identify highly uncertain structures. The ML-predicted IR spectra are obtained from three independent MLMD trajectories, each initiated with different velocity seeds and propagated using forces obtained by averaging predictions from a committee of models. First, 5 ps of each trajectory is used for thermalization, while the following 50 ps are used for the IR simulation.

For all MD simulations, a time step of 0.5 fs is used, and, unless specified otherwise, the temperature is set to 300 K.

### Infrared spectra calculation

The IR spectrum is calculated from an MD trajectory by computing the auto-correlation function of the time derivative of the dipole moment, $\dot{\mu }$, according to the following equation^17^:

1 $${I}_{IR}(\omega )\propto \mathop{\int}\nolimits_{\!{-\infty}}^{+\infty }{\left\langle \dot{\mu }\left(\tau \right)\dot{\mu }\left(\tau +t\right)\right\rangle }_{\tau }{e}^{-i\omega t}dt.$$

In this work, all auto-correlation functions are computed using the Wiener–Khinchin theorem^95^. A Hann window function^96^ and zero-padding are applied to the auto-correlation functions before the Fourier transform to obtain high-quality IR spectra. A maximum correlation depth of 1000 fs is used. For IR spectra processing, we utilized the autocorrelation function from SchNetPack^97^ and integrated it into our workflow.

### Spectra preprocessing and similarity measures

To enable accurate comparison between experimental and theoretical IR spectra, it is crucial to correct for artifacts present in the data. A common issue in experimental spectra is baseline correction, which can obscure spectral features and hinder meaningful analysis. We address this by automating baseline correction using the method described in ref. ^77^, as illustrated in Fig. S10 of the Supplementary Information. Additionally, to reconcile differences in frequency sampling between experimental and theoretical spectra, we apply linear interpolation to map the theoretical data onto the experimental frequency grid.

For comparison between theories or between theory and experiment, we utilize two metrics to evaluate the degree of correlation between the spectra: Pearson’s correlation coefficient (PCC) (Eq. 2) and the Wasserstein distance (WD) (Eq. 3), both implemented in scikit-learn 1.5.2^98^. The PCC is defined as:

2 $$\,{\text{PCC}}\,=\frac{\mathop{\sum }\nolimits_{i = 1}^{n}({x}_{i}-{\bar{x}})({y}_{i}-{\bar{y}})}{\sqrt{\mathop{\sum }\nolimits_{{i} = 1}^{{n}}{({x}_{i}-{\bar{x}})}^{2}}\sqrt{\mathop{\sum }\nolimits_{{i} = 1}^{n}{({y}_{i}-{\bar{y}})}^{2}}},$$

where *x*_*i*_ and *y*_*i*_ represent the intensities of the respective spectra, while $\bar{x}$ and $\bar{y}$ are their mean values^77,99,100^.

The WD is defined in terms of an integral, rather than the discrete definition of PCC:

3 $$WD(\mu ,\nu )=\mathop{\inf }\limits_{\gamma \in \Gamma (\mu ,\nu )}{\int}_{\!{\mathbb{R}}\times {\mathbb{R}}}| | x-y| | \,d\gamma (x,y),$$

where *μ* and *ν* are the distributions corresponding to the two spectra being compared. *Γ*(*μ*, *ν*) denotes the set of all joint distributions with marginals *μ* and *ν*, ∣∣*x* − *y*∣∣ is the distance between points sampled from the distributions, and *dγ*(*x*, *y*) represents the infinitesimal mass transported from *x* to *y*^77,101^.

## Supplementary information

Supplementary information

## Data Availability

To facilitate open materials science ^102^, the reference DFT-based AIMD dataset is accessible at 10.5281/zenodo.14657903, while the data curated through the active learning scheme is available at 10.5281/zenodo.14699673. Additionaly, the spectral data published in this study can be found in ref. ^67^, along with a tutorial on how to obtain IR spectra from the trained models.
