# Supplementary material for: Leveraging active learning-enhanced machine-learned interatomic potential for efficient infrared spectra prediction
Source: NPJ Comput Mater. 2025 Oct 29;11(1):324. doi: 10.1038/s41524-025-01827-8 (PMC12571885; doi:10.1038/s41524-025-01827-8)
Supplement: Supplementary file 1 — Supplementary information [file 41524_2025_1827_MOESM1_ESM.pdf]

Supplementary information for: Leveraging active learning-enhanced machine-learned interatomic potential for efficient infrared spectra prediction

Nitik Bhatia[1,2], Patrick Rinke[1,2,3,4], and Ondřej Krejčí[2,5]

[1]*Department of Physics, Technical University of Munich, James-Franck-Strasse 1, Garching, 85748, Germany*
[2]*Department of Applied Physics, Aalto University, P.O. Box 11000, AALTO, FI-00076, Finland*
[3]*Atomistic Modelling Center, Munich Data Science Institute, Technical University of Munich, Walther-Von-Dyck Str. 10, Garching, 85748, Germany*
[4]*Munich Center for Machine Learning (MCML), Munich, Germany*
[5]*Department of Mechanical and Materials Engineering, Vesilinnantie 5, Turku, Finland*

September 4, 2025

Corresponding author: patrick.rinke@tum.de

Content

Figure S1 – A set of 24 representative small organic molecules used in this study. Figure S2 – Energy distribution of methanol, comparing DFT-based AIMD data and active learning data relative to the optimized structure. Figure S3 – Spectral similarity for 24 molecules, comparing Exp.-DFT, DFT-ML, and Exp.-ML using PCC and WD. Figure S4 – IR spectra of methanol at five temperatures, with DFT-ML similarity evaluated at each temperature using PCC and WD. Figure S5 – IR spectra of ethanol at five temperatures, with DFT-ML similarity evaluated at each temperature using PCC and WD. Figure S6 – Spectral similarity for eight organic molecules, comparing Exp.-ML spectra using PCC and WD. Figure S7 – Harmonic IR spectra of four molecules comparing DFT and ML model predictions. Figure S8 – Comparison of IR spectra for methanol and ethanol computed using Experimental data, DFT based AIMD, and ML models (MACE_PALIRS, MACE_off23, and MACE_off24). Figure S9 – Visualization of normal modes of methanol and sampling of structures along them. Figure S10 – Baseline correction applied to the experimental spectrum, showing improved alignment with ML predictions.

Table S1 – MAE and RMSE for energies, forces, and dipole moments for 8 molecules used for testing the transferability of the trained models, shown in Fig S6. Table S2 – Harmonic IR spectra of four molecules comparing DFT and ML model predictions. Table S3 – Similarity results for methanol and ethanol molecules, expressed as PCC and WD, comparing Experimental spectra from NIST, DFT-based AIMD, and ML models (MACE_PALIRS, MACE_off23, and MACE_off24) corresponding to Figure S8.

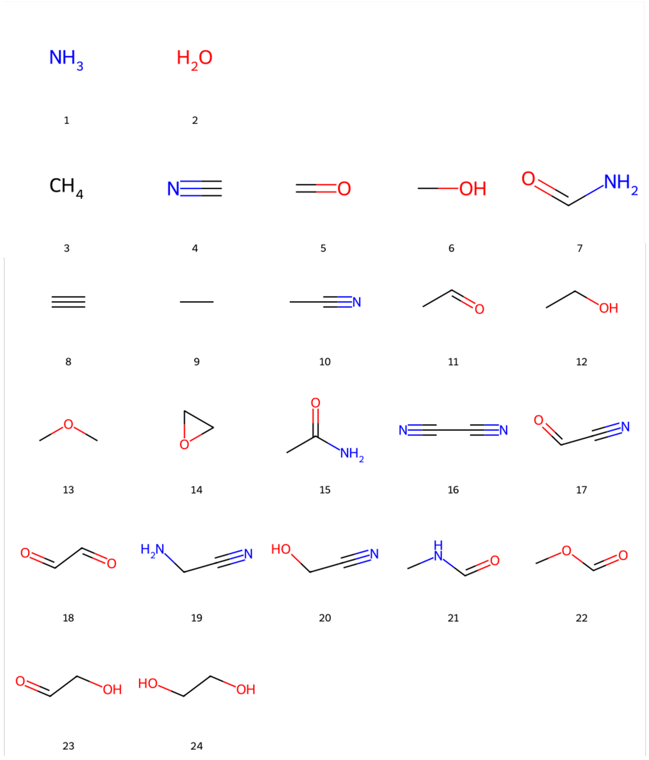

Figure S1: Visualization of the 24 small organic molecules included in this study.

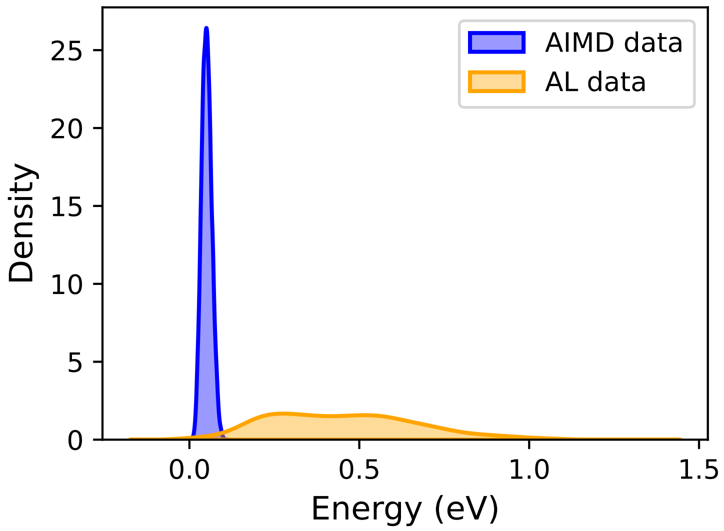

Figure S2: Energy distribution of the methanol molecule, with energies relative to the optimized structure. The data includes AIMD data (40,000 samples from DFT-based AIMD simulations) and AL data (664 samples obtained via active learning)

.

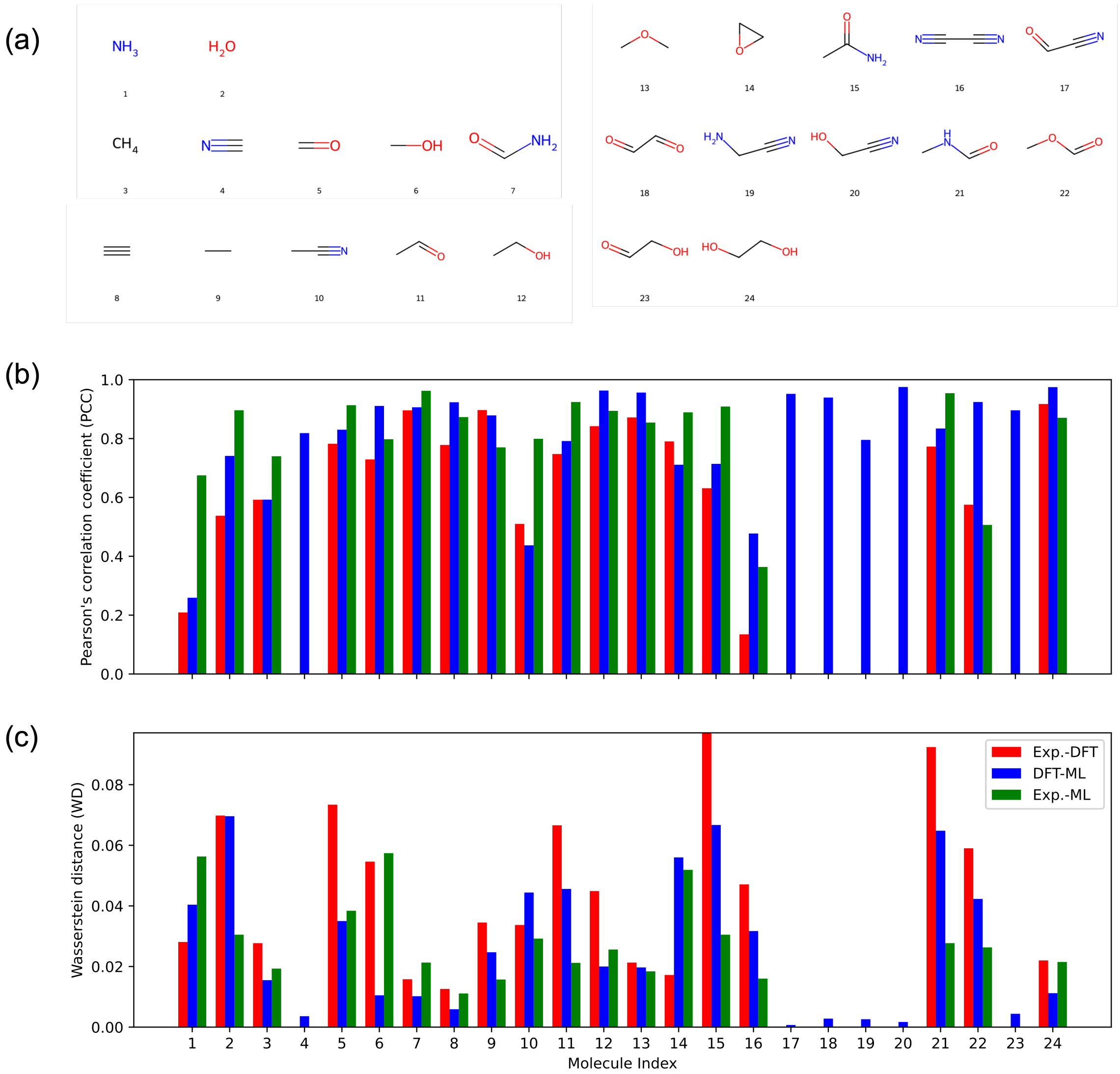

Figure S3: Spectral similarity for (a) all 24 organic molecules, comparing the pairs Exp.-DFT, DFT-ML, and Exp.-ML using (b) Pearson's correlation coefficient (PCC) and (c) Wasserstein distance (WD). For six molecules (indices 4, 17, 18, 19, 20, and 23), experimental spectra are unavailable, so only the DFT-ML comparison is provided for these cases.

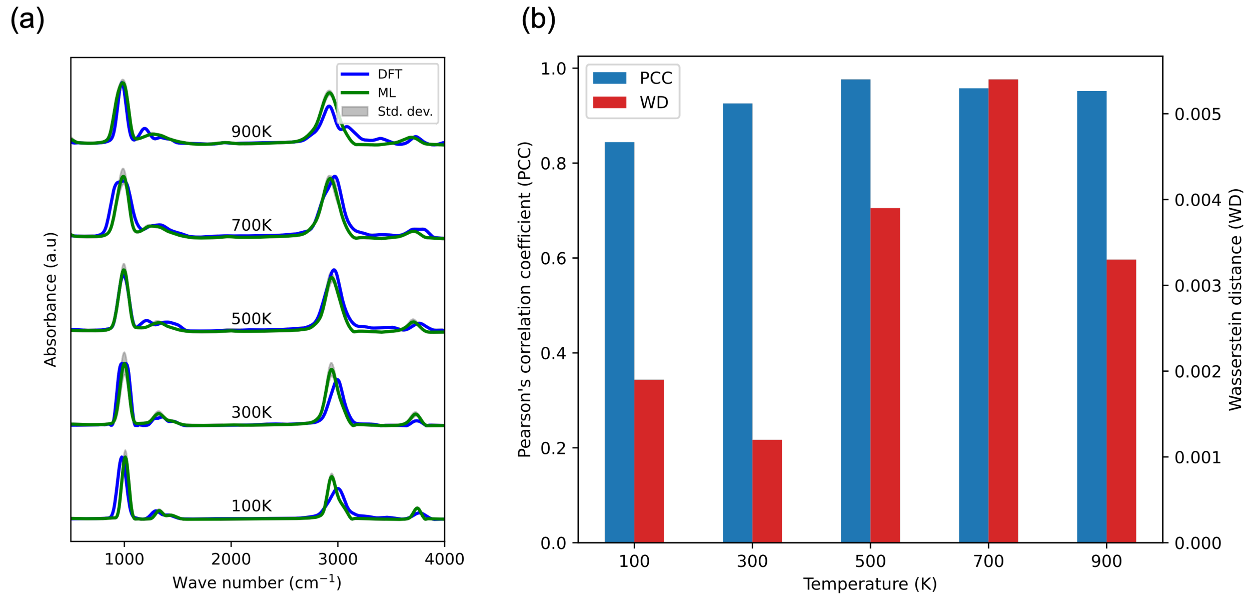

Figure S4: (a) IR spectra of methanol at five different temperatures: 100K, 300K, 500K, 700K, and 900K. (b) Spectral similarity at each temperature between DFT-ML predictions, evaluated using Pearson's correlation coefficient (PCC) and Wasserstein distance (WD).

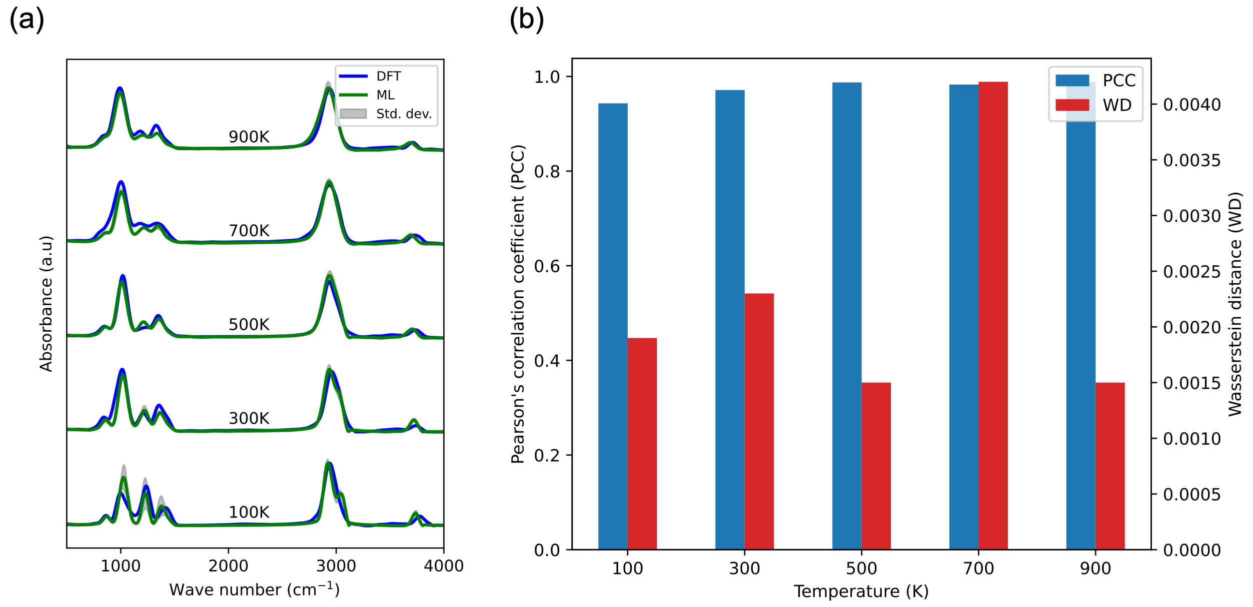

Figure S5: (a) IR spectra of ethanol at five different temperatures: 100K, 300K, 500K, 700K, and 900K. (b) Spectral similarity at each temperature between DFT-ML predictions, evaluated using Pearson's correlation coefficient (PCC) and Wasserstein distance (WD).

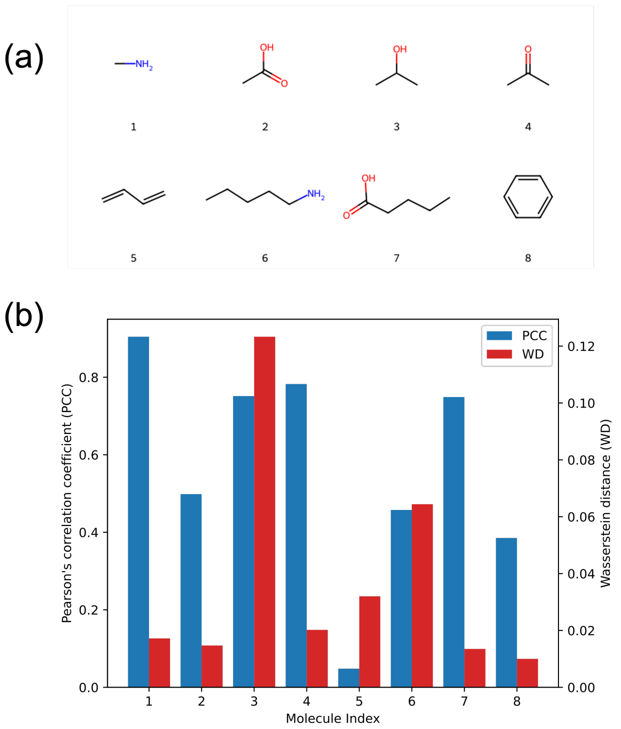

Figure S6: Spectral similarity for (a) eight organic molecules, distinct from the training data or featuring increasing carbon atoms and varying functional groups, (b) evaluated between Exp.-ML predictions using Pearson's correlation coefficient (PCC) and Wasserstein distance (WD).

Table S1: MAE and RMSE for energies, forces, and dipole moments for 8 molecules used for testing the transferability of the trained models, shown in Fig S6 (a). This test set was generated the same way as the test set for, described in the Methods section of the main text, just with 50 structures per molecule.

Molecule	Energy (eV)		Forces (eV/Å)		Dipole moment (Debye)	
	MAE	RMSE	MAE	RMSE	MAE	RMSE
1. Methyl amine	0.207	0.207	0.045	0.067	0.071	0.082
2. Acetic acid	0.554	0.558	0.168	0.263	0.243	0.279
3. Isopropyl alcohol	0.101	0.105	0.084	0.130	0.042	0.054
4. Acetone	0.025	0.030	0.072	0.109	0.138	0.156
5. Butadiene	1.844	1.887	0.721	1.119	0.119	0.152
6. Pentanamine	0.355	0.359	0.124	0.179	0.118	0.144
7. Pentanoic acid	0.824	0.827	0.131	0.183	0.307	0.365
8. Benzene	5.176	5.185	0.588	0.799	0.089	0.115

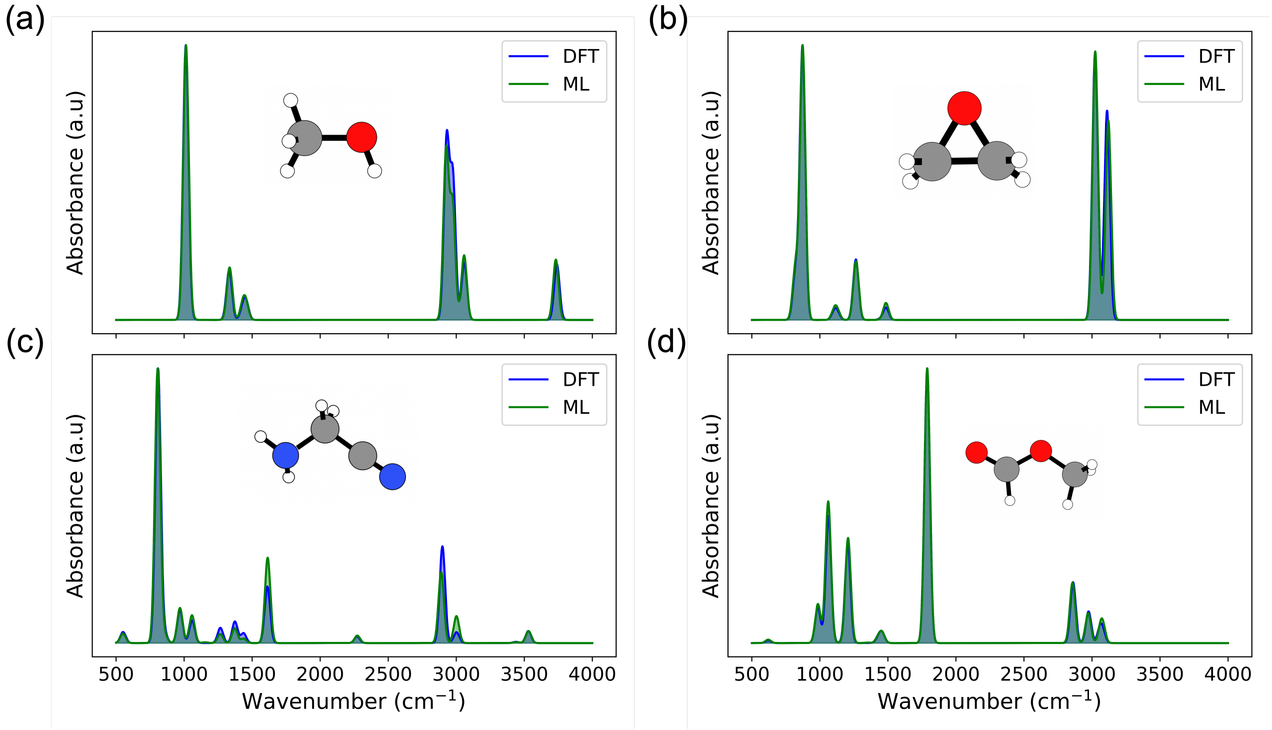

Figure S7: Harmonic IR spectra of four molecules comparing DFT and ML model predictions: (a) Molecule index 6, (b) Molecule index 14, (c) Molecule index 19, and (d) Molecule index 22. The molecule indices correspond to Fig. S1.

Table S2: Errors in harmonic frequency (cm^{-1}) and intensity (D/Å)^2 amu^{-1} between ML-predicted and DFT spectra for the 24 molecules in the training set. The molecule indices correspond to Fig. S1.

Molecule	Frequency (cm^{-1})		Intensity (D/Å)^2 amu^{-1}	
	MAE	RMSE	MAE	RMSE
1	2.27	2.70	0.0075	0.0131
2	4.30	5.58	0.0129	0.0152
3	1.69	2.20	0.0267	0.0459
4	4.13	5.79	0.0232	0.0376
5	7.35	8.50	0.0484	0.0741
6	3.72	6.26	0.0585	0.0967
7	2.97	4.13	0.0588	0.0855
8	11.18	11.89	0.0310	0.0652
9	2.46	3.07	0.0533	0.1031
10	1.69	1.93	0.0277	0.0470
11	2.81	3.99	0.0745	0.1023
12	3.88	5.89	0.0974	0.1482
13	3.49	5.75	0.1956	0.3827
14	1.97	2.98	0.0348	0.0649
15	9.31	19.33	0.1283	0.1699
16	2.52	2.85	0.0026	0.0062
17	3.92	5.89	0.0482	0.0740
18	3.99	5.79	0.0095	0.0170
19	1.73	2.66	0.0780	0.1269
20	3.86	5.44	0.2377	0.4963
21	2.64	4.11	0.0882	0.1435
22	2.50	3.59	0.0764	0.1396
23	4.95	6.80	0.1518	0.2062
24	10.41	28.86	0.3087	1.1398
Average error	4.23	10.42	0.103	0.360

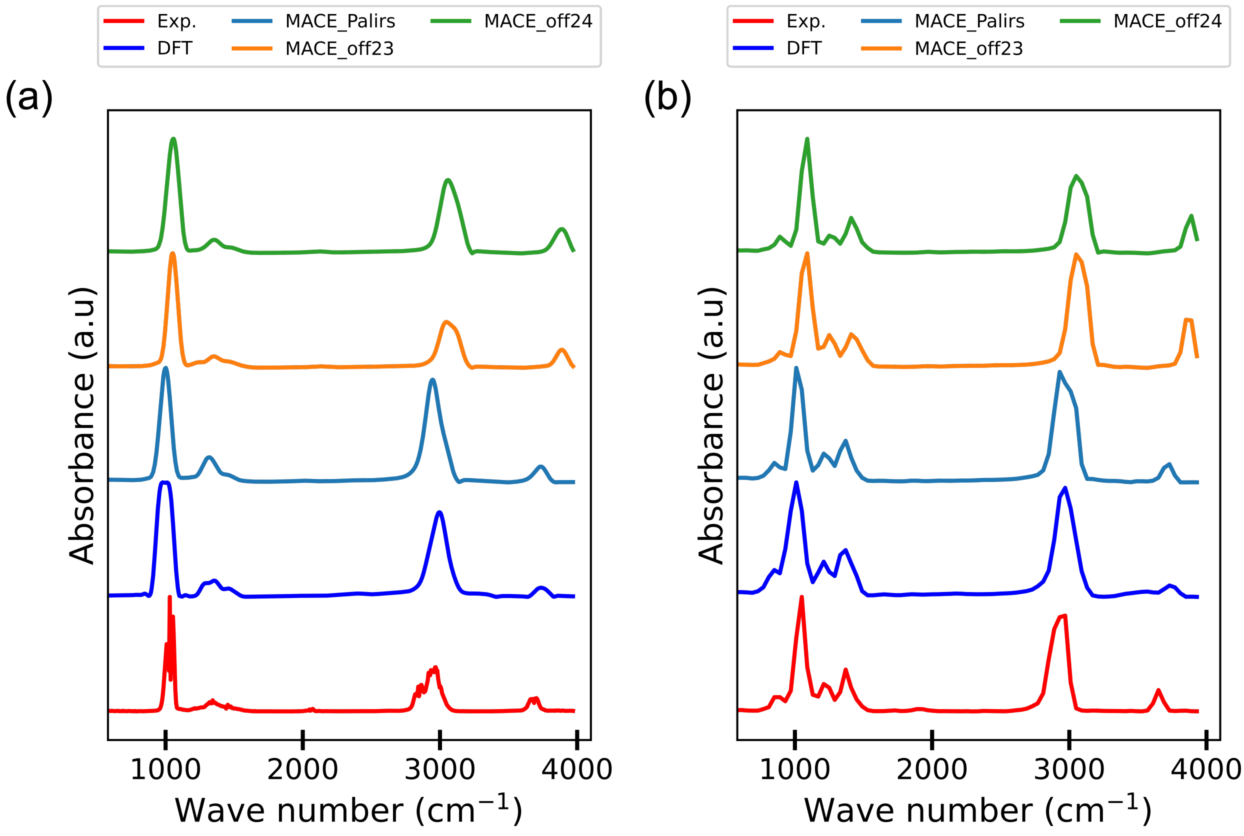

Figure S8: Comparison of IR spectra for methanol (a) and ethanol (b) computed using Experimental data, DFT based AIMD, and ML models (MACE_PALIRS, MACE_off23, and MACE_off24).
Note: Here, DFT refers to calculations at the PBE [1] level, combined with the Tkatchenko–Scheffler treatment of van der Waals interactions [2] and "light" basis sets as implemented in the FHI-AIMS package and used in this work. The MACE-off [3] models are trained on the SPICE dataset [4], computed at the ωB97M-D3(BJ)/def2-TZVPPD level of quantum mechanics, as implemented in the PSI4 software package.

Table S3: Similarity results for methanol and ethanol molecules, expressed as Pearson's correlation coefficient (PCC) and Wasserstein distance (WD), comparing Experimental spectra from NIST, DFT-based AIMD, and ML models (MACE_PALIRS, MACE_off23, and MACE_off24) corresponding to Figure S8.

Molecule	Comparison	Methanol		Ethanol	
		PCC	WD	PCC	WD
Experimental vs DFT		0.7291	0.0546	0.8421	0.0449
Experimental vs ML	MACE_PALIRS	0.7902	0.0550	0.8722	0.0290
	MACE_off23	0.6548	0.0239	0.3832	0.0347
	MACE_off24	0.5759	0.0447	0.4164	0.0168
DFT vs ML	MACE_PALIRS	0.9149	0.0099	0.9666	0.0211
	MACE_off23	0.6152	0.0308	0.4942	0.0151
	MACE_off24	0.6094	0.0139	0.4824	0.0400

Note: Here, DFT refers to calculations at the PBE [1] level, combined with the Tkatchenko–Scheffler treatment of van der Waals interactions [2] and "light" basis sets as implemented in the FHI-AIMS package and used in this work. The MACE-off [3] models are trained on the SPICE dataset [4], computed at the ωB97M-D3(BJ)/def2-TZVPPD level of quantum mechanics, as implemented in the PSI4 software package.

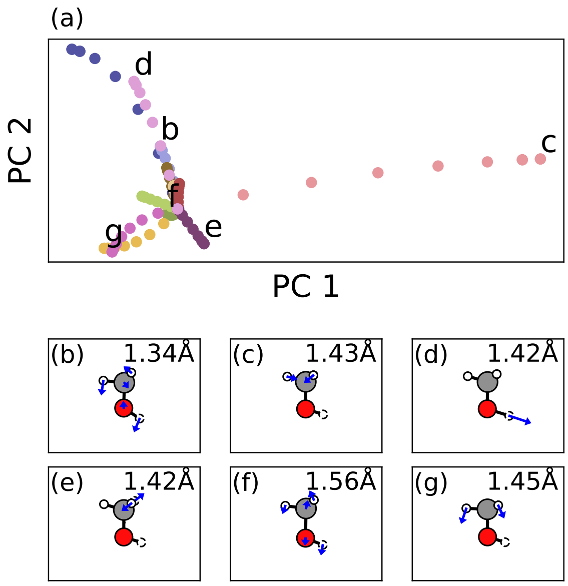

Figure S9: (a) Principal component analysis (PCA) on global Smooth Overlap of Atomic Positions [5,6] (SOAP) features of methanol structures. The filled circles in the same color are the structures along one specific mode and different color indicates different modes. (b)-(g) Snapshots of the selected structures. The normal vectors are plotted in blue arrows and C-O bond length is labeled. The visualization was carried out using plotting code adapted from Z. Tang *et al.* [7].

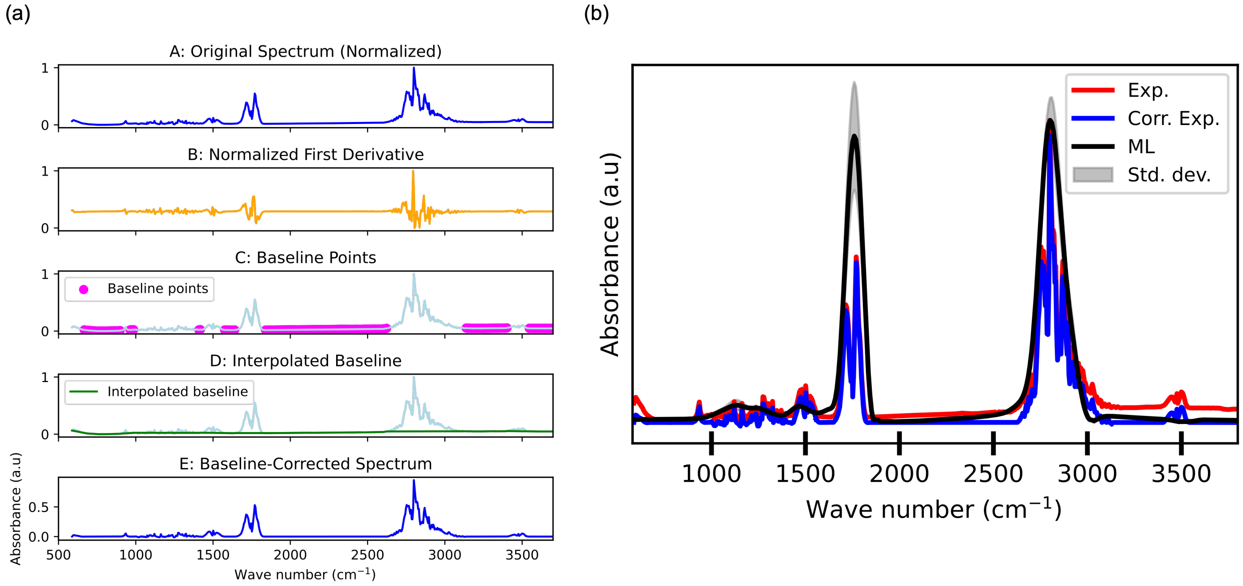

Figure S10: (a) Baseline correction procedure [8] for the IR spectrum of formaldehyde. The method involves several steps, starting from (A) Original IR spectrum of formaldehyde. (B) First derivative of the spectrum. Data points where derivative is exceeding a threshold (0.0008) and their neighboring points are identified and excluded from baseline points labeling. (C) Remaining data points used for baseline fitting are marked in magenta. (D) Linear interpolation (green line) is used to create the baseline. (E) Final baseline-corrected spectrum after subtraction of the baseline.

(b) Comparison of the original experimental IR spectrum, the baseline-corrected spectrum, and the ML-predicted spectrum for formaldehyde. The baseline correction removes distortions, enhancing the alignment between the experimental and ML spectra.

References

[1] John P. Perdew, Kieron Burke, and Matthias Ernzerhof. Generalized Gradient Approximation Made Simple [Phys. Rev. Lett. 77, 3865 (1996)]. *Physical Review Letters*, 78(7):1396–1396, February 1997.

[2] Alexandre Tkatchenko and Matthias Scheffler. Accurate Molecular Van Der Waals Interactions from Ground-State Electron Density and Free-Atom Reference Data. *Physical Review Letters*, 102(7):073005, February 2009.

[3] Dávid Péter Kovács, J. Harry Moore, Nicholas J. Browning, Ilyes Batatia, Joshua T. Horton, Yixuan Pu, Venkat Kapil, William C. Witt, Ioan-Bogdan Magdău, Daniel J. Cole, and Gábor Csányi. Mace-off: Short-range transferable machine learning force fields for organic molecules. *Journal of the American Chemical Society*, 147(21):17598–17611, 2025.

[4] Peter Eastman, Pavan Kumar Behara, David L. Dotson, Raimondas Galvelis, John E. Herr, Josh T. Horton, Yuezhi Mao, John D. Chodera, Benjamin P. Pritchard, Yuanqing Wang, Gianni De Fabritiis, and Thomas E. Markland. Spice, a dataset of drug-like molecules and peptides for training machine learning potentials. *Scientific Data*, 10(1):11, Jan 2023.

[5] Sandip De, Albert P. Bartók, Gábor Csányi, and Michele Ceriotti. Comparing molecules and solids across structural and alchemical space. *Phys. Chem. Chem. Phys.*, 18:13754–13769, 2016.

[6] Lauri Himanen, Marc O.J. Jäger, Eiaki V. Morooka, Filippo Federici Canova, Yashasvi S. Ranawat, David Z. Gao, Patrick Rinke, and Adam S. Foster. Dscribe: Library of descriptors for machine learning in materials science. *Computer Physics Communications*, 247:106949, 2020.

[7] Zeyuan Tang, Stefan T. Bromley, and Bjørk Hammer. A machine learning potential for simulating infrared spectra of nanosilicate clusters. *The Journal of Chemical Physics*, 158(22):224108, June 2023.

[8] Beatriz von der Esch, Laurens D. M. Peters, Lena Sauerland, and Christian Ochsenfeld. Quantitative Comparison of Experimental and Computed IR-Spectra Extracted from Ab Initio Molecular Dynamics. *Journal of Chemical Theory and Computation*, 17(2):985–995, 2021.
